# Effects of Water and Energy on Plant Diversity along the Aridity Gradient across Dryland in China

**DOI:** 10.3390/plants10040636

**Published:** 2021-03-27

**Authors:** Shuran Yao, Muhammad Adnan Akram, Weigang Hu, Yuan Sun, Ying Sun, Yan Deng, Jinzhi Ran, Jianming Deng

**Affiliations:** 1 State Key Laboratory of Grassland Agro-Ecosystem, School of Life Sciences, Lanzhou University, Lanzhou 730000, China; yaoshr15@lzu.edu.cn (S.Y.); ynmadnan@gmail.com (M.A.A.); huwg@lzu.edu.cn (W.H.); suny16@lzu.edu.cn (Y.S.); ysun18@lzu.edu.cn (Y.S.); yvette456ding@163.com (Y.D.); dengjm@lzu.edu.cn (J.D.); 2 State College of Forestry, Southwest Forestry University, Bailongsi 300, Kunming 650224, China

**Keywords:** aridity gradient, dryland, plant diversity, plant lifeforms, water–energy dynamics

## Abstract

Plants need water and energy for their growth and reproduction. However, how water and energy availability influence dryland plant diversity along the aridity gradient in water-limited regions is still lacking. Hence, quantitative analyses were conducted to evaluate the relative importance of water and energy to dryland plant diversity based on 1039 quadrats across 184 sites in China’s dryland. The results indicated that water availability and the water–energy interaction were pivotal to plant diversity in the entire dryland and consistent with the predictions of the water–energy dynamic hypothesis. The predominance of water limitation on dryland plant diversity showed a weak trend with decreasing aridity, while the effects of energy on plants were found to be significant in mesic regions. Moreover, the responses of different plant lifeforms to water and energy were found to vary along the aridity gradient. In conclusion, the study will enrich the limited knowledge about the effects of water and energy on plant diversity (overall plants and different lifeforms) in the dryland of China along the aridity gradient.

## 1. Introduction

The growth and reproduction of plants are dependent on water availability and environmental energy [1,2]. Environmental energy (heat) is the basic requirement of all plants needed to perform the different functions and activities of their lives, such as photosynthesis. Water is an integral part of plant tissues and without it, plants cannot perform many physiological activities [3,4]. Environmental energy and water are interrelated [3]; for example, energy can increase the water-use efficiency of plants to promote photosynthesis, as snow can reduce the freezing effect on plants [1,5]. However, excessively high or low energy may change the water into vapor or ice, and these physical processes could result in the limitation of a plant’s growth and development [6,7]. In contrast, when energy is abundant, water restriction (e.g., limiting water metabolic processes and closing of the stomata) also prevents plants from using the energy for photosynthesis [8,9,10].

The effects of water and energy on constraining the physicochemical and physiological processes of plants might generate and maintain a broad gradient of plant diversity [1,6,11,12,13]. The water–energy dynamic hypothesis emphasizes that the large-scale gradient of species diversity is related to both water and energy [11,13,14,15]. It provides theoretical support to understand the environmental capacity to support greater plant-species richness [6,16]. The relative importance of water and energy maintenance to plant diversity may depend on climatic regions [17,18]. For example, water is a limiting factor in warm regions, where the energy is sufficient; while energy becomes more important in cold regions, where inputs of energy are low [15]. Conversely, the richness–energy relationship depends on water availability [6]. Species diversity also has shown the strong effects of energy and water on woody plant species [1]. Nevertheless, how water and energy availability interact to influence dryland plant diversity in local communities along the water gradient in water-limited regions is still an open question.

The United Nations Environmental Program (UNEP) defines drylands as arid regions where the aridity index (the ratio of annual precipitation to annual potential evapotranspiration) is less than 0.65 [19]. Based on the aridity index, drylands can be classified as dry-subhumid regions, semiarid regions, arid regions, and hyperarid regions [20,21]. Approximately 45% of Earth’s land-surface area is arid [22]. In China’s dryland, ephemeral plants, annual herbs, perennial herbs, and woody plants (including shrubs, sub-shrubs, and small trees) are the dominant plant lifeforms, but tall trees are rare. Different lifeforms have distinct physiological attributes and growth forms [23,24,25]. For example, ephemeral plants are a special group of dryland herbaceous plants that typically depend on transient precipitation and temperature, consequently growing fast and adapting to the dry environment [23,24]. Shrubs have a strong rooting system to effectively extract moisture in low-moisture environments [2,26]. Different lifeforms have distinct adaptive strategies to survive in severe environmental conditions [25], which may lead to their different responses to water and energy along the aridity gradient.

In this study, 184 sites were surveyed and 1039 quadrats were sampled along the aridity gradient in the entire dryland (including hyperarid, arid, semiarid, and dry-subhumid regions) of China. The objectives of the present study were: (1) to assess the relative importance of water and energy to overall plant diversity along the aridity gradient; and (2) to evaluate whether the effects of water and energy on plant diversity of different lifeforms vary along the aridity gradient. In the light of distinct adaptive strategies of different plant lifeforms, it is predicted that water and energy might influence the lifeform diversity differently.

## 2. Materials and Methods

### 2.1. Study Region

**Figure 1:**
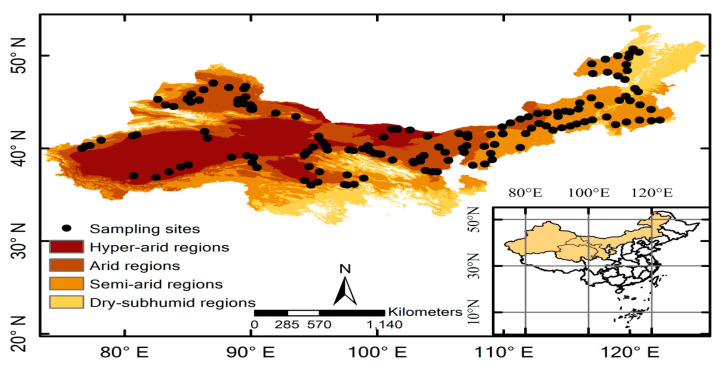
Map of vegetation sampling sites based on the different aridity regions. Generated with ArcMap 10.3 (ESRI, Redlands, CA, USA).

The region is located in the northwestern dryland of China (36.01°~50.70° N and 76.62°~122.40° E) and occupies approximately 2.44 million square kilometers (Figure 1).

The climate of the study region is characterized by a dry temperate continental climate with relatively low precipitation (ranging from 25 to 485 mm) and strong temperature heterogeneity (mean annual temperature ranging from −3.79 to 12.64 ℃) (Table S1). The whole area spans hyperarid to dry-subhumid regions. These long-term drought differences have formed different plant communities and unique vegetation landscapes. From hyperarid regions to dry-subhumid regions, the vegetation types in study regions range from typically temperate desert vegetation to temperate desert steppe to temperate arid steppe.

### 2.2. Sampling Survey

At each site, vegetation was surveyed using the nested sampling design. The nested samples were started from 0.5 m × 0.5 m for grassland vegetation types, or 1 m × 1 m for desert vegetation types (layer by layer) to sample until 30 m × 30 m, or 100 m × 100 m by nested smaller quadrats within larger quadrats. For example, 10 m × 10 m quadrats contained a nested subquadrat of 5 m × 5 m, and the 5 m × 5 m subquadrat contained a further nested subquadrat of 1 m × 1 m. To reduce the area-effect on species diversity, the quadrat of 30 m × 30 m scale was used at each study site. In each quadrat, the scientific name of every plant was noted and species identification was conducted by taxonomists. At each site, we divided all plants into different plant lifeforms such as annual herbs, perennial herbs, and woody plants (including shrubs, sub-shrubs, and small trees) according to the stem texture and lifespan of the plants, following the Flora Reipublicae Popularis Sinicae (http://www.iplant.cn/frps, accessed on 5 December 2020).

In dryland, there are a special group of herbs called “ephemeral plants” that tend to grow fast, thereby completing their life cycles faster than other herbaceous plants [23,24,27]. In temperate desert ecosystems, spring ephemerals play an important role in the community structure [28,29]. They are characterized by specific biological traits that helped them to adjust and adapt to deserts [30]. As compared to other plants such as annual and perennial herbs, ephemerals grow very fast and show greater light-use efficiency, and allocate a greater percentage of their biomass to reproduction [30,31]. Spring ephemerals’ aboveground phenology is adapted to a narrow window of favorable environmental conditions, and they maximize photosynthetic gain by taking advantage of intense light [32,33]. They accelerate leaf senescence with warmer spring temperatures [34]. Ephemeral sampling is difficult because of their short lifespan and limited above-ground activity period [33]. Moreover, spring ephemerals are extremely sensitive to climate changes and grow only in favorable environmental conditions (in the presence of adequate moisture) [31].

In this study, ephemeral plants were referred to as spring ephemeral plants, which germinate in early spring and generally complete their life cycle in 60–70 days (before the arrival of summer drought, dying before or in July) [27,28,35,36,37]. Therefore, according to their life history, these special plants were distinguished from the other long-lived herbaceous plants, following the Flora Reipublicae Popularis Sinicae (http://www.iplant.cn/frps, accessed on 5 December 2020). From 2013 to 2017 (sampling was carried out from early June to late July), in total, 184 sites were surveyed and 1039 quadrats were sampled for plant species during the growing season.

### 2.3. Environmental Factors

According to the water–energy dynamic hypothesis, water refers to the variables representing precipitation, and energy refers to the variables representing heat [13]. Many studies also often use annual potential evapotranspiration (PET, mm) and annual actual evapotranspiration (AET, mm) to represent energy and actual water consumption, respectively [12,17,38]. Therefore, the frequently used water and energy variables were selected to assess the relative importance of water and energy on dryland plant diversity. The water variables included mean annual precipitation (MAP, mm), annual actual evapotranspiration (AET, mm), precipitation of the wettest quarter (PWQ, mm), and precipitation of the driest quarter (PDQ, mm). The energy variables were mean annual temperature (MAT, °C), annual potential evapotranspiration (PET, mm), mean temperature of warmest quarter (MTWQ, °C), and mean temperature of coldest quarter (MTCQ, °C). MAP, PWQ, PDQ, MAT, MTWQ, and MTCQ were taken from the WorldClim (http://worldclim.org/version2, accessed on 31 July 2017) [39]; and AET, PET, and the aridity index (i.e., AI= annual precipitation/annual potential evapotranspiration) were extracted from the Consortium for Spatial Information (CGIAR-CSI) (http://www.cgiar-csi.org/data, accessed on 31 July 2017). All variables were derived at a resolution of 30 s × 30 s (ca 1 km × 1 km at the equator). The mean values and range of the environmental variables are shown in Table S1.

### 2.4. Data Analysis

First, the aridity index (AI) was used to divide the study area into four different regions, including hyperarid regions (aridity index <0.05), arid regions (aridity index from 0.05 to <0.2), semiarid regions (aridity index from 0.2 to <0.5), and dry-subhumid regions (aridity index from 0.5 to <0.65) [19]. The location map was generated with ArcMap 10.3 (ESRI, Redlands, CA, USA).

Then, a principal components analysis (PCA) was used for water and energy variables to eliminate collinearity among variables (Pearson correlation coefficients >0.7, see Table S2). The major water (water PC) and energy gradients (energy PC) were extracted according to a broken-stick stopping rule [1].

To evaluate the relationship between plant diversity and water availability or environmental energy along the aridity gradient, linear mixed-effects models were used to model the plant diversity (represented by the number of plant species) considering water and energy variables. The linear mixed-effects models and PCA were conducted in R using the “lme4” and “vegan” packages, respectively. To eliminate the potential annual variability on plants that may result from sampling in different years, “year” as a random intercept and “water” and “energy” as fixed effects were used in the models. To obtain a nearly normal distribution, the plant diversity was transformed by taking its square root. Nonlinear models such as quadratic models were used for a better fit relative to the linear models via the Akaike information criterion (AIC). The models with minimal AIC and the regression significance, which were tested by F- and T-tests, respectively, were selected. Theoretically, the effect of energy on species diversity is unimodal because of the water effect [11,13], so the quadratic terms with a negative sign were accepted. Partial residual regression was also used to obtain an estimate of the effects of water availability or environmental energy on plant diversity that was not confounded by environmental energy or water availability.

Variation partitioning [40] was used to estimate the common and unique contribution of water availability and environmental energy to the variation in plant diversity. Moreover, variation partitioning was also used to divide the total variation of plant diversity into three different parts (independent components, covarying effects between water and energy, and unexplained variability) to reveal the impact of change in water and energy for plant diversity. All the statistical analyses were carried out in R software (version 3.6.0) [41].

## 3. Results

### 3.1. Water, Energy, and Plant Diversity in Dryland

Based on the principal components analysis, the first principal components (PC1) had the maximum proportion of variance for both water (76.50%) and energy groups (80.57%) (Table 1). In this study, the correlations among water variables and among energy variables were found to be positive (Table S2). Moreover, the water and energy variables had positive loadings on the PC1 (Table 1), which suggested that water availability and environmental energy increased with the increasing value of PC1. Therefore, the first principal components were selected to represent the major water (water PC1) and energy gradients (energy PC1) (Table 1).

**Figure 2:**
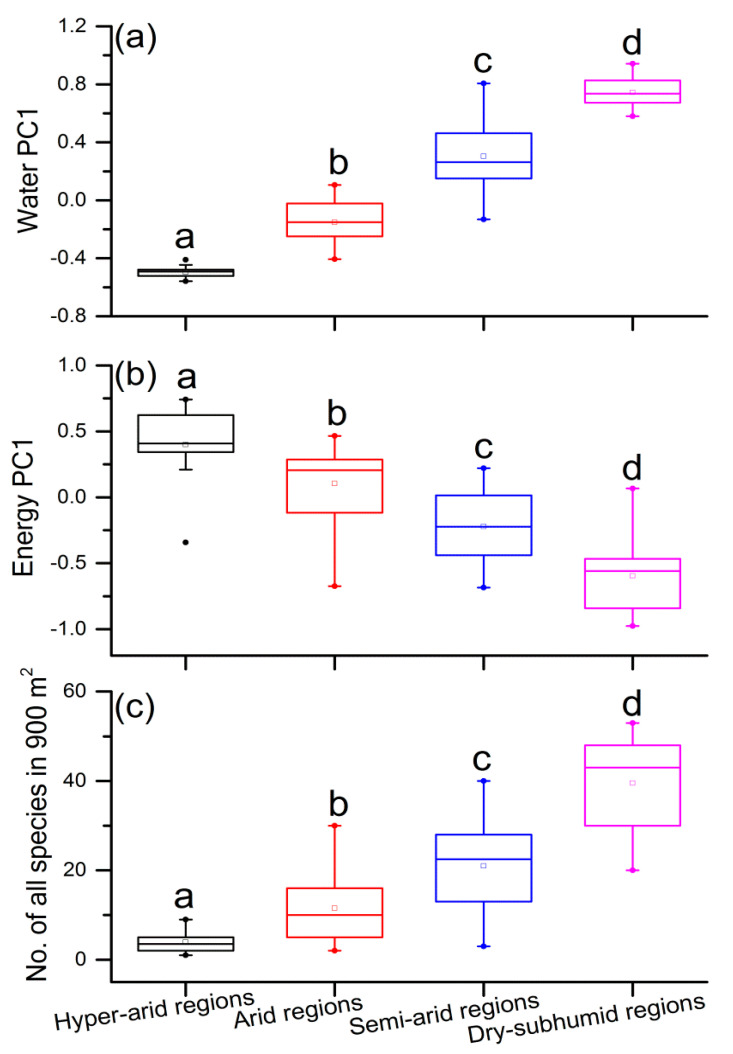
Boxplots for (**a**) water, (**b**) energy, and (**c**) plant diversity (number of all species in 900 m^2^ in each site) based on the different aridity regions (hyperarid regions: n = 26 sites; arid regions: n = 91 sites; semiarid regions: n = 50 sites; dry-subhumid regions: n = 17 sites). Regions with the different letters are significantly different from each other at *p* < 0.05 by one-way ANOVA.

Water availability significantly increased from hyperarid regions to dry-subhumid regions (Figure 2a), but the opposite trend was noted for environmental energy (Figure 2b). The plant diversity of dryland in 900 m^2^ of quadrats significantly increased as the aridity lessened across the studied regions (Figure 2c).

### 3.2. Relative Importance of Water and Energy on Overall Plant Diversity along the Aridity Gradient

**Figure 3:**
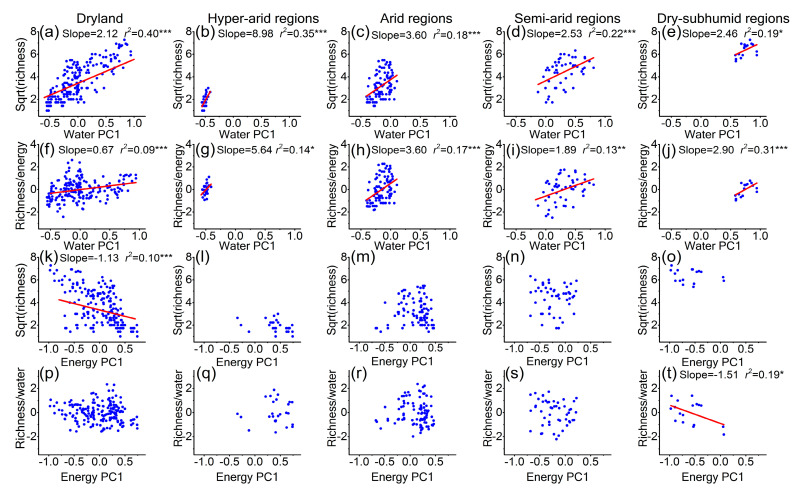
The relationship between (**a–e**) overall plant diversity and water; (**f–j**) plant diversity conditioned on energy (richness/energy) and water; (**k–o**) overall plant diversity and energy; (**p–t**) plant diversity conditioned on water (richness/water) and energy across the entire dryland in China and the different aridity regions. Only significant trend lines (*p* < 0.05) are fixed. Plant diversity is expressed as the number of species in 900 m^2^ in each site and is square-rooted in models, and *r*^2^ is the adjusted coefficient of determination. Dryland: n = 184 sites; hyperarid regions: n = 26 sites; arid regions: n = 91 sites; semiarid regions: n = 50 sites; dry-subhumid regions: n = 17 sites.

**Figure 4:**
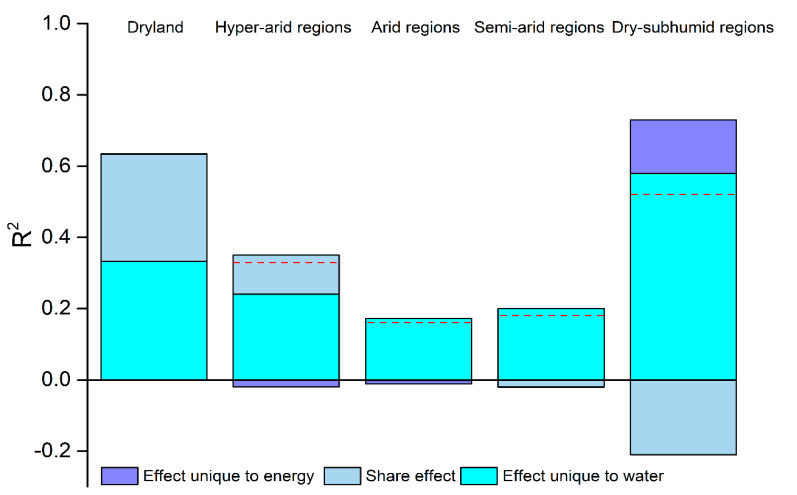
The variation of water and energy effects on overall plant diversity in China’s dryland by variation partitioning. Effect unique to energy is the explained variability by the independent components of environmental energy. Share effect represents variability explained by the covarying component of water and energy. Effect unique to water is the explained variability by the independent components of water. Negative effects indicate that explained variation on plant diversity is lowered by the energy unique effect or the interaction between water and energy in different aridity regions, and the red dashed line expresses the actual total variation explained by water and energy in different aridity regions. Plant diversity is expressed as the number of species in 900 m^2^ in each site and is square-rooted in models. R^2^ is the adjusted coefficient of determination. Dryland: n = 184 sites; hyperarid regions: n = 26 sites; arid regions: n = 91 sites; semiarid regions: n = 50 sites; dry-subhumid regions: n = 17 sites.

In the entire dryland, the diversity of overall plants increased with the increase of water PC1 (*r*^2^ = 0.40; *p* < 0.001), while it decreased with energy PC1 (*r*^2^ = 0.10; *p* < 0.001) (Figure 3a,k). However, when the effect of energy was controlled in partial residual regressions, the relationship of plant diversity and water remained significant and positive, but the variation in diversity explained by water was strongly reduced (*r*^2^ only was 0.09) (Figure 3f). Moreover, the shared effect of water and energy explained 30% of the variation in plant diversity in the entire dryland (Figure 4). More interestingly, the effects of energy on plant diversity were absent when the effects of water were controlled (Figure 3p).

In different arid regions, the relationship of plant diversity and water also kept its trends with water PC1 (Figure 3b–e), as well as in partial residual regressions (Figure 3g–j). However, the slope was decreased (from 9.0 to 2.5) with the decrease in aridity (Figure 3b–e). Energy did not show any effects on plant diversity in each arid region (Figure 3l–o). However, a negative effect of energy was seen on plant diversity after the removal of the water effect in the dry-subhumid regions (Figure 3t).

### 3.3. The Responses of Different Plant Lifeforms to Water and Energy along the Aridity Gradient

**Figure 5:**
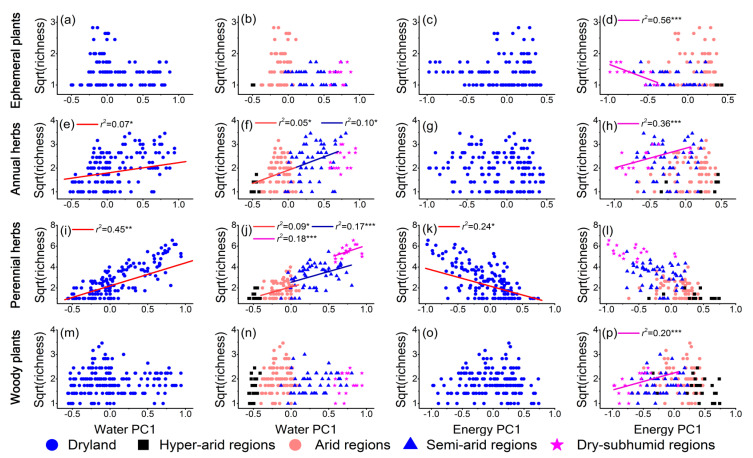
The relationship of plant diversity among different plant life-forms and water, energy across the entire dryland in China and the different aridity regions. Only significant trend lines (*p* < 0.05) are shown. Plant diversity is expressed as the number of species in 900 m^2^ in each site and is square-rooted in models, and *r*^2^ is the adjusted coefficient of determination. (**a–d**) Ephemeral plants (dryland: n = 105 sites; hyperarid regions: n = 3 sites; arid regions: n = 54 sites; semiarid regions: n = 35 sites; dry-subhumid regions: n = 13 sites). (**e–h**) Annual herbs (dryland: n = 144 sites; hyperarid regions: n = 7 sites; arid regions: n = 73 sites; semiarid regions: n = 47 sites; dry-subhumid regions: n = 17 sites). (**i–l**) Perennial herbs (dryland: n = 149 sites; hyperarid regions: n =15 sites; arid regions: n = 68 sites; semiarid regions: n = 49 sites; dry-subhumid regions: n = 17 sites). (**m–p**) Woody plants (dryland: n =180 sites; hyperarid regions: n = 24 sites; arid regions: n = 89 sites; semiarid regions: n = 50 sites; dry-subhumid regions: n = 17 sites).

**Figure 6:**
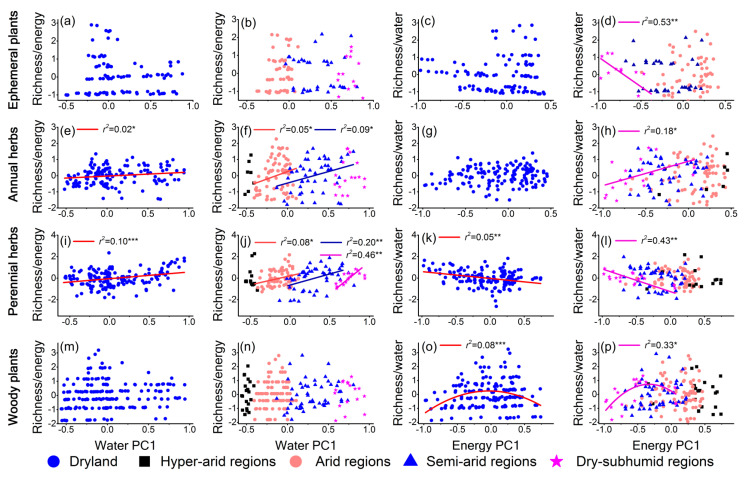
The relationship of the residuals with water and energy across the entire dryland in China and the different aridity regions. Richness/energy represents the residuals of the model fit to diversity as a function of water. Richness/water represents plant diversity conditioned on energy. Only significant trend lines (*p* < 0.05) are shown, and *r*^2^ is the adjusted coefficient of determination. (**a–d**) Ephemeral plants (dryland: n = 105 sites; hyperarid regions: n = 3 sites; arid regions: n = 54 sites; semiarid regions: n = 35 sites; dry-subhumid regions: n = 13 sites). (**e–h**) Annual herbs (dryland: n = 144 sites; hyperarid regions: n = 7 sites; arid regions: n = 73 sites; semiarid regions: n = 47 sites; dry-subhumid regions: n = 17 sites). (**i–l**) Perennial herbs (dryland: n = 149 sites; hyperarid regions: n =15 sites; arid regions: n = 68 sites; semiarid regions: n = 49 sites; dry-subhumid regions: n = 17 sites). (**m–p**) Woody plants (dryland: n =180 sites; hyperarid regions: n = 24 sites; arid regions: n = 89 sites; semiarid regions: n = 50 sites; dry-subhumid regions: n = 17 sites).

The effects of water and energy on the diversity of different lifeforms varied along the aridity gradient (Figure 5 and Figure 6, and Figure S1). Specifically, the influences of water and energy on plant diversity for ephemeral plants were not found to be significant, except in the dry-subhumid regions, where the energy showed a negative effect on plant diversity (Figure 5a–d). Water played a key role in the plant diversity of annual herbs and perennial herbs in the entire dryland and most arid regions (Figure 5e,f,i,j). However, in the dry-subhumid regions, regardless of whether the water or energy effects were removed, energy exhibited a significant positive effect on plant diversity for annual herbs (Figure 5h and Figure 6h), while water limitation was not found significant (Figure 5f and Figure 6f).

For perennial herbs, water explained the greater variation in plant diversity in the dry-subhumid regions (*r*^2^ was 0.46) when the effect of energy was controlled as compared with the uncontrolled effect of energy (*r*^2^ was 0.18) (Figure 5j and Figure 6j). Moreover, the negative effects of energy became significant in the dry-subhumid regions when the effects of water were controlled (Figure 6l). Water had no significant contributions to the plant diversity of woody plants (Figure 5m,n), and the diversity–water relationships still were not prominent when the effect of energy was controlled (Figure 6m,n). However, the diversity–energy unimodal relationship of woody plants became significant in the entire dryland after the removal of the water effect (Figure 6o). As in the case of other lifeforms, energy also had a noteworthy influence on woody plant diversity in the dry-subhumid regions (Figure 5p and Figure 6p). Furthermore, there were similar results to those of the partial residual regressions when the variation-partitioning analyses were used (Figure S1).

## 4. Discussion

### 4.1. The Effects of Water-Energy Dynamics on Dryland Plant Diversity

Among the numerous environmental factors, water and energy are considered to be the two most important factors affecting the pattern of plant diversity [11]. The results indicated that water is a main limiting resource in dryland plant communities (Figure 3) [40,42]. Energy is less important than water as a driver of both broad-scale and fine-scale plant diversity in the dryland of China (Figure 3). Moreover, the study revealed that the effects of energy on plant diversity were mainly shared with water in dryland (Figure 4) because high energy (such as high temperature) can enhance the rates of evapotranspiration [17]. This is why a strong negative relationship was observed between plant diversity and energy in the entire dryland (Figure 3k), which suggests that high temperature restricted the plant diversity in areas with strong water deficits [1,6]. This analysis of dryland plant diversity in China has followed the water–energy dynamic hypothesis, and can be used to explain the broad-scale plant diversity in temperate drylands (Figure 4) [11,13,14,15].

However, the strong limitations of water on plants make it difficult to assess the specific effect of energy on plant diversity in dryland. In this study, when the water effect on plant diversity was controlled by partial residual regressions, the diversity–energy relationships were not found to be significant in the entire dryland and the regions with severe water limitations (Figure 3q–s). In contrast, the influence of water on plant diversity became stronger with increasing aridity (Figure 3b–e) [1], which suggests that drought was a critical limitation of dryland plant diversity. Based on these observations, it can be predicted that water deficits drove physiological damage (such as injuring plant cells and inhibiting photosynthetic rate, etc.) that may have much stronger control of plants than energy in the dryland of China [10]. Plant evolutionary history of diversification and speciation can provide an explanation for this observation [43]. Earlier studies on plant diversification and speciation in the dryland of China make it clear that aridification could promote plant diversification and speciation in temperate deserts and steppes [43,44,45]. In addition to habitat fragmentation, desert expansion and environmental aridification also could cause population isolation of plant species, potentially promoting the diversification and speciation of desert plant species [44,46].

### 4.2. The Different Responses of Plant Lifeforms to Water and Energy along the Aridity Gradient

As predicted, the effects of water and energy on plant diversity were different among plant lifeforms. The results showed that the influence of water on ephemeral plant diversity was not significant (Figure 5a,b and Figure 6a,b), which can be attributed to the characteristics of the ephemeral plants that complete their life cycle in short periods (by reproducing and growing very fast) [23]. Moreover, ephemeral plants are largely distributed in the Gurbantunggut Desert, where the snowmelt and melting of glaciers from mountains (like the Tianshan Mountains) are the main water sources for plant germination and growth in the spring season [47]. The herbaceous plants (after removing ephemeral plants; i.e., annual herbs and perennial herbs in this study) were greatly affected by water limitation in dryland (Figure 5 and Figure 6). Specifically, the study analyses indicated that the effect of water limitation on perennial herbs was stronger than that on annual herbs, which can be explained by their different reproductive strategies [48,49]. In addition, in the hyperarid regions, water had no significant effect on the plant diversity of each lifeform. Of course, hyperarid regions are characterized by extreme drought with very little precipitation. The survival of herbaceous plants may mainly benefit from the microhabitats (with higher soil fertility and increased water availability) created by woody plants [50,51] because woody plants have deeper root systems to obtain groundwater [2,23]. This is also the explanation for the weak effect of water on woody plant diversity.

The results indicated that the influence of energy on plant diversity often became important in dry-subhumid regions where water limitations are low for dryland (Figure 5 and Figure 6). Notably, the responses of distinct plant lifeforms to energy varied (Figure 6). For ephemeral plants, the significant effect of energy on their diversity was not considered, because the only three species were in the sites of dry-subhumid regions. For the varied effects of energy on plant diversity for annual and perennial herbs, this phenomenon can be explained by the vertical structure of the community. In this study, the vegetation type in dry-subhumid regions was the meadow steppe (Hulunber Meadow Steppe), which was dominated by the perennial graminoid [52]. The temperature in this area was extremely high and low in summer and winter, respectively [52]. Perennial herbs, mostly in the upper part of the communities, can receive more energy (heat) than annual herbs, which mostly create the lower layer. Too much energy (a high temperature in summer) means that tissue damage is reinforced, photosynthetic activity is inhibited, and growth is limited for perennial herbs [8,9]. Conversely, provision of energy for annual herbs was likely not sufficient for their growth. Therefore, the response of perennial herbs was negative to energy, while the response of annual herbs was positive. Indeed, a study on the effects of warming on plant diversity in grassland detected a significant reduction of diversity of the perennial herbs, while diversity of annual herbs increased with warming [53].

The effects of low temperature on herbaceous plants were excluded in accordance with our preliminary yet unpublished study about distribution and determinants of plants in this region. It has shown that the cold temperature in winter does not affect herbaceous plants, while it has a unimodal relationship only with woody plants [54]. This may be due to winter snow that can protect the underground parts of perennial herbs by weakening the effects of soil freezing [5]. However, the aboveground parts of woody plants must be frost-tolerant. A study on plant phylogeography in arid Northwest China has shown that major Quaternary glaciations were absent in the region, and the plant species can tolerate extreme cold [43]. In summary, these findings show that growth strategies have significant effects on the response of plant lifeforms to water–energy dynamics.

In addition, although human influence was not included in this study, we believe that it had no perverse effects on the results. As far as possible, the samples were collected from the sites with relatively little human disturbance (e.g., enclosed grasslands and nature reserves, etc.) to minimize human impact. Second, some studies have shown that grazing reduces vegetation cover to a low extent only, and does not significantly reduce the number of perennial herbs [55,56]. Most important of all, these results indicated that the effect of water became stronger when the effect of energy was controlled in the dry-subhumid regions (Figure 5j and Figure 6j), which means that energy itself must have had a significantly negative influence on the plant diversity of perennial herbs, compared with the effect of water. Certainly, in addition to water and energy, many other factors also may affect plant diversity in the dryland. For instance, soil factors, anthropogenic activities, climatic variables, and different interspecific interactions are all likely to influence large-scale plant diversity patterns. This could be reflected in unexplained parts of the variation partitioning in this study, but this was not the main focus of the present study. Future research can further explore the effects of these factors.

## 5. Conclusions

This study demonstrated that the broad-scale patterns of dryland plant diversity depend mainly on the effects of water availability and the water–energy interaction. However, the water-limitation effects on plants were found to be much stronger than energy in the severe drought regions and decreased along with aridity, while the effects of energy on plant diversity stood out in the dry-subhumid regions. The effects of water and energy varied in different plant lifeforms because of distinct growth strategies. Moreover, this study supports the water–energy dynamic hypothesis and highlights the relative importance of water and energy availability in determining plant diversity of overall plant and different lifeforms in the dryland of China. These findings will be a benefit to conservation planning for reducing effects of future climate change on the plants in the dryland of China.

## Figures and Tables

**Table 1:** The loadings, proportion, and cumulative proportion of the first four principal components for the principal component analysis (PCA) of water and energy predictors.

Climatic Variables	PC 1	PC 2	PC 3	PC 4
**Water**				
Annual precipitation (mm)	0.568	−0.079	0.387	0.722
Annual actual evapotranspiration (mm)	0.563	−0.069	−0.823	−0.009
Precipitation of wettest quarter (mm)	0.552	−0.259	0.406	−0.681
Precipitation of the driest quarter (mm)	0.236	0.960	0.083	−0.125
Proportion explained	76.50%	22.47%	0.95%	0.08%
Cumulative proportion	76.50%	98.97%	99.92%	100.00%
**Energy**				
Mean annual temperature (°C)	0.554	0.006	−0.321	−0.768
Annual potential evapotranspiration (mm)	0.538	0.030	0.842	0.037
Mean temperature of warmest quarter (°C)	0.435	−0.748	−0.270	0.421
Mean temperature of coldest quarter (°C)	0.463	0.662	−0.340	0.481
Proportion explained	80.57%	17.02%	2.33%	0.08%
Cumulative proportion	80.57%	97.59%	99.92%	100.00%

## Data Availability

The climatic variables MAP, PWQ, PDQ, MAT, MTWQ, and MTCQ data were obtained from the WorldClim (http://worldclim.org/version2, accessed on 5 December 2020); and AET, PET, and the aridity index data were extracted from the Consortium for Spatial Information (CGIAR-CSI) (http://www.cgiar-csi.org/data, accessed on 5 December 2020).

## Supplementary Materials

The following materials are available online at https://www.mdpi.com/article/10.3390/plants10040636/s1, Table S1: Summary of the environmental variables examined in this study, Table S2: Correlation matrix among various water and energy variables at the study sites, Figure S1: The variation of water and energy effects on plant diversity for lifeforms by variation partitioning. The left value is the explained variability by the independent components of water, the middle value represents variability explained by the covarying component of water and energy, and the right value is the explained variability by the independent components of the environment. Residuals represent unexplained variability. All of the variation values were adjusted by coefficient of determination. In Figure S1b, NaN represents the only three sites for ephemeral plants in hyperarid regions, so it is not operable by variation partitioning.

## References

[1] Xu X., Wang Z., Rahbek C., Sanders N.J., Fang J. (2016). Geographical variation in the importance of water and energy for oak diversity. J. Biogeogr..

[2] Hageer Y., Esperón-Rodríguez M., Baumgartner J.B., Beaumont L.J. (2017). Climate, soil or both? Which variables are better predictors of the distributions of Australian shrub species?. PeerJ.

[3] Clarke A., Gaston K.J. (2006). Climate, energy and diversity. Proc. R. Soc. B Biol. Sci..

[4] Deng J.M., Wang G.X., Morris E.C., Wei X.P., Li D.X., Chen B.M., Zhao C.M., Liu J., Wang Y. (2006). Plant mass-density relationship along a moisture gradient in north-west China. J. Ecol..

[5] Groffman P.M., Driscoll C.T., Fahey T.J., Hardy J.P., Fitzhugh R.D., Tierney G.L. (2001). Colder soils in a warmer world: A snow manipulation study in a northern hardwood forest ecosystem. Biogeochemistry.

[6] Francis A.P., Currie D.J. (2003). A Globally Consistent Richness-Climate Relationship for Angiosperms. Am. Nat..

[7] O’Brien E.M. (1993). Climatic Gradients in Woody Plant Species Richness: Towards an Explanation Based on an Analysis of Southern Africa’s Woody Flora. J. Biogeogr..

[8] Vico G., Thompson S.E., Manzoni S., Molini A., Albertson J.D., Almeida-Cortez J.S., Fay P.A., Feng X., Guswa A.J., Liu H. (2015). Climatic, ecophysiological, and phenological controls on plant ecohydrological strategies in seasonally dry ecosystems. Ecohydrology.

[9] Deng J.-M., Li T., Wang G.-X., Liu J., Yu Z.-L., Zhao C.-M., Ji M.-F., Zhang Q., Liu J.-q. (2008). Trade-offs between the metabolic rate and population density of plants. PLoS ONE.

[10] Forni C., Duca D., Glick B.R. (2017). Mechanisms of plant response to salt and drought stress and their alteration by rhizobacteria. Plant Soil.

[11] O’Brien E.M. (1998). Water-energy dynamics, climate, and prediction of woody plant species richness: An interim general model. J. Biogeogr..

[12] Zhang C., Cai D., Li W., Guo S., Guan Y., Bian X., Yao W. (2017). Effect of the Long-Term Mean and the Temporal Stability of Water-Energy Dynamics on China’s Terrestrial Species Richness. Int. J. Geo-Inf..

[13] O’Brien E.M. (2006). Biological relativity to water–energy dynamics. J. Biogeogr..

[14] O’Brien E.M., Field R., Whittaker R.J. (2000). Climatic gradients in woody plant (tree and shrub) diversity: Water-energy dynamics, residual variation, and topography. Oikos.

[15] Hawkins B.A., Field R., Cornell H.V., Currie D.J., Guégan J.-F., Kaufman D.M., Kerr J.T., Mittelbach G.G., Oberdorff T., O’Brien E.M. (2003). Energy, water, and broad-scale geographic patterns of species richness. Ecology.

[16] Panda R.M., Behera M.D., Roy P.S., Biradar C. (2017). Energy determines broad pattern of plant distribution in Western Himalaya. Ecol. Evol..

[17] Kreft H., Jetz W. (2007). Global patterns and determinants of vascular plant diversity. Proc. Natl. Acad. Sci. USA.

[18] Currie D.J., Mittelbach G.G., Cornell H.V., Field R., Guégan J.F., Hawkins B.A., Kaufman D.M., Kerr J.T., Oberdorff T., O’Brien E. (2004). Predictions and tests of climate-based hypotheses of broad-scale variation in taxonomic richness. Ecol. Lett..

[19] UNEP (1992). World Atlas of Desertification.

[20] Feng S., Fu Q. (2013). Expansion of global drylands under a warming climate. Atmos. Chem. Phys..

[21] Huang J., Li Y., Fu C., Chen F., Fu Q., Dai A., Shinoda M., Ma Z., Guo W., Li Z. (2017). Dryland climate change: Recent progress and challenges. Rev. Geophys..

[22] Prăvălie R. (2016). Drylands extent and environmental issues. A global approach. Earth-Sci. Rev..

[23] Chen R., Ran J., Huang H., Dong L., Sun Y., Ji M., Hu W., Yao S., Lu J., Gong H. (2019). Life history strategies drive size-dependent biomass allocation patterns of dryland ephemerals and shrubs. Ecosphere.

[24] Waudby H.P., Petit S. (2015). Ephemeral plant indicators of livestock grazing in arid rangelands during wet conditions. Rangel. J..

[25] Akram M.A., Wang X., Hu W., Xiong J., Zhang Y., Deng Y., Ran J., Deng J. (2020). Convergent Variations in the Leaf Traits of Desert Plants. Plants.

[26] Turner R.M., Bowers J.E., Burgess T.L. (2005). Sonoran Desert Plants.

[27] Jia Y., Sun Y., Zhang T., Shi Z., Maimaitiaili B., Tian C., Feng G. (2020). Elevated precipitation alters the community structure of spring ephemerals by changing dominant species density in Central Asia. Ecol. Evol..

[28] Huang G., Li Y., Padilla F.M. (2015). Ephemeral plants mediate responses of ecosystem carbon exchange to increased precipitation in a temperate desert. Agric. For. Meteorol..

[29] Angert A., Huxman T., Barron-Gafford G., Gerst K., Venable D. (2007). Linking growth strategies to long-term population dynamics in a guild of desert annuals. J. Ecol..

[30] Yuan S., Tang H. (2010). Patterns of ephemeral plant communities and their adaptations to temperature and precipitation regimes in Dzungaria Desert, Xinjiang. Biodivers. Sci..

[31] Chen Y., Zhang L., Shi X., Liu H., Zhang D. (2019). Life history responses of two ephemeral plant species to increased precipitation and nitrogen in the Gurbantunggut Desert. PeerJ.

[32] Rothstein D.E., Zak D.R. (2001). Photosynthetic adaptation and acclimation to exploit seasonal periods of direct irradiance in three temperate, deciduous-forest herbs. Funct. Ecol..

[33] Augspurger C.K., Salk C.F. (2017). Constraints of cold and shade on the phenology of spring ephemeral herb species. J. Ecol..

[34] Lapointe L., Lerat S. (2006). Annual growth of the spring ephemeral Erythronium americanum as a function of temperature and mycorrhizal status. Botany.

[35] Qiu Y., Liu T., Zhang C., Liu B., Pan B., Wu S., Chen X. (2018). Mapping Spring Ephemeral Plants in Northern Xinjiang, China. Sustainability.

[36] Fan L.-L., Tang L.-S., Wu L.-F., Ma J., Li Y. (2014). The limited role of snow water in the growth and development of ephemeral plants in a cold desert. J. Veg. Sci..

[37] Zhang L. (2002). Ephemeral plants in Xinjiang (III): Significance of community and resources. J. Plant.

[38] Li L., Wang Z., Zerbe S., Abdusalih N., Tang Z., Ma M., Yin L., Mohammat A., Han W., Fang J. (2013). Species richness patterns and water-energy dynamics in the drylands of Northwest China. PLoS ONE.

[39] Fick S.E., Hijmans R.J. (2017). WorldClim 2: New 1-km spatial resolution climate surfaces for global land areas. Int. J. Climatol..

[40] Palpurina S., Wagner V., von Wehrden H., Hájek M., Horsák M., Brinkert A., Hölzel N., Wesche K., Kamp J., Hájková P. (2017). The relationship between plant species richness and soil pH vanishes with increasing aridity across Eurasian dry grasslands. Glob. Ecol. Biogeogr..

[41] Team R.D.C. (2019). Version 3.6.0: A Language and Environment for Statistical Computing.

[42] Speziale K.L., Ruggiero A., Ezcurra C. (2010). Plant species richness–environment relationships across the Subantarctic–Patagonian transition zone. J. Biogeogr..

[43] Meng H.-H., Gao X.-Y., Huang J.-F., Zhang M.-L. (2015). Plant phylogeography in arid Northwest China: Retrospectives and perspectives. J. Syst. Evol..

[44] Su Z., Zhang M. (2013). Evolutionary response to Quaternary climate aridification and oscillations in north-western China revealed by chloroplast phylogeography of the desert shrub Nitraria sphaerocarpa (Nitrariaceae). Biol. J. Linn. Soc..

[45] Gao X.-Y., Meng H.-H., Zhang M.-L. (2014). Diversification and vicariance of desert plants: Evidence inferred from chloroplast DNA sequence variation of *Lagochilus ilicifolius* (Lamiaceae). Biochem. Syst. Ecol..

[46] Wang Q., Abbott R.J., Yu Q.S., Lin K., Liu J.Q. (2013). Pleistocene climate change and the origin of two desert plant species, *Pugionium cornutum* and *Pugionium dolabratum* (*Brassi-caceae*), in northwest China. New Phytol..

[47] Fang X.-W., Turner N.C., Palta J.A., Yu M.-X., Gao T.-P., Li F.-M. (2014). The distribution of four Caragana species is related to their differential responses to drought stress. Plant Ecol..

[48] Harrison S.P., Gornish E.S., Copeland S. (2015). Climate-driven diversity loss in a grassland community. Proc. Natl. Acad. Sci. USA.

[49] Rabotnov T. (1969). On coenopopulations of perennial herbaceous plants in natural coenoses. Vegetatio.

[50] Ochoa-Hueso R., Eldridge D.J., Delgado-Baquerizo M., Soliveres S., Bowker M.A., Gross N., Le Bagousse-Pinguet Y., Quero J.L., García-Gómez M., Valencia E. (2018). Soil fungal abundance and plant functional traits drive fertile island formation in global drylands. J. Ecol..

[51] Saiz H., Gómez-Gardeñes J., Borda J.P., Maestre F.T. (2018). The structure of plant spatial association networks is linked to plant diversity in global drylands. J. Ecol..

[52] Yan R., Tang H., Xin X., Chen B., Murray P.J., Yan Y., Wang X., Yang G. (2016). Grazing intensity and driving factors affect soil nitrous oxide fluxes during the growing seasons in the Hulunber meadow steppe of China. Environ. Res. Lett..

[53] Klein J.A., Harte J., Zhao X.Q. (2004). Experimental warming causes large and rapid species loss, dampened by simulated grazing, on the Tibetan Plateau. Ecol. Lett..

[54] Yao S., Hu W., Ji M., Dong L., Deng J. Distribution, determinants, and relative importance of different plant life-forms across drylands in China.

[55] Rutherford M.C., Powrie L.W. (2011). Can heavy grazing on communal land elevate plant species richness levels in the Grassland Biome of South Africa?. Plant Ecol..

[56] Yan R., Xin X., Yan Y., Wang X., Zhang B., Yang G., Liu S., Deng Y., Li L. (2015). Impacts of differing grazing rates on canopy structure and species composition in Hulunber meadow steppe. Rangel. Ecol. Manag..

