# Supplementary material for: Effects of Water and Energy on Plant Diversity along the Aridity Gradient across Dryland in China
Source: Plants (Basel). 2021 Mar 27;10(4):636. doi: 10.3390/plants10040636 (PMC8067162; doi:10.3390/plants10040636)
Supplement: Supplementary file 1 [file plants-10-00636-s001.pdf]

Effects of Water and Energy on Plant Diversity along the Aridity Gradient across Dryland in China

Shuran Yao [1,†], Muhammad Adnan Akram [1,†], Weigang Hu [1], Yuan Sun [1], Ying Sun [1], Yan Deng [1,2], Jinzhi Ran [1,*] and Jianming Deng [1]

[1] State Key Laboratory of Grassland Agro-Ecosystem, School of Life Sciences, Lanzhou University, Lanzhou, Gansu 730000, China; yaoshr15@lzu.edu.cn (S.Y.); ynmadnan@gmail.com (M.A.A.); huwg@lzu.edu.cn (W.H.); suny16@lzu.edu.cn (Y.S.); ysun18@lzu.edu.cn (Y.S.)

[2] State College of Forestry, Southwest Forestry University, Bailongsi 300, Kunming 650224, China; yvette456ding@163.com (Y.D.)

* Correspondence: ranjz@lzu.edu.cn (J.R.)

† These authors contributed equally to this manuscript.

Supplementary Information

Table S1. Summary of the environmental variables examined in this study.

Table S2. Correlation matrix among various water and energy variables at the study sites.

Figure S1. The variation of water and energy effects on plant diversity for life-forms by variation partitioning. The left value is the explained variability by the independent components of water; the middle value represents variability explained by the co-varying component of water and energy, the right value is the explained variability by the independent components of environmental. Residuals are representing unexplained variability. All of the variation values are adjusted by coefficient of determination. In Figure S1 (b): NaN represents the only three sites for ephemeral plants in hyper-arid regions, so it is not operable by variation partitioning.

Table S1. Summary of the environmental variables examined in this study.

Climatic variables	Dryland		Hyper-arid regions		Arid regions		Semi-arid regions		Dry-subhumid regions	
	Mean	Range	Mean	Range	Mean	Range	Mean	Range	Mean	Range
**Water**										
Annual precipitation(mm)	181.68	25-485	41.15	25-62	134.43	55-221	267.66	126-443	396.71	345-485
Annual actual evapotranspiration(mm)	984.35	706-1363	1176.08	887-1363	1018.18	755-1147	892.36	760-1051	780.65	706-939
Precipitation of wettest quarter(mm)	115.07	19-329	28.15	19-47	74.05	37-135	179.78	62-306	277.24	240-329
Precipitation of the driest quarter(mm)	7.02	0-22	1.00	0-3	8.14	0-22	7.18	2-20	9.76	7-14
**Energy**										
Mean annual temperature(℃)	5.77	(-3.79)-12.64	8.94	2.53-12.64	6.86	(-0.68)-10.83	3.97	(-0.75)-8.08	0.34	(-3.79)-7.09
Annual potential evapotranspiration(mm)	167.30	23-400	39.85	23-60	120.91	64-184	253.78	163-358	356.24	315-400
Mean temperature of warmest quarter(℃)	21.01	11.28-28.77	23.31	14.76-26.35	21.76	11.28-28.77	19.45	11.65-22.95	18.03	16.05-22.74
Mean temperature of coldest quarter(℃)	-11.24	(-26.37)-(-2.74)	-6.98	(-10.36)-(-2.75)	-9.85	(-17.83)-(-3.51)	-13.18	(-22.11)-(-5.91)	-19.46	(-26.37)-(-10.23)

Table S2. Correlation matrix among various water and energy variables at the study sites.

	MTWQ	MTCQ	PET	PDQ	MAP	PWQ	AET
**MAT**	0.781***	0.837***	0.935***	-0.197**	-0.569***	-0.588***	-0.622***
**MTWQ**		0.321***	0.719***	0.152*	-0.341***	-0.392***	-0.409***
**MTCQ**			0.789***	-0.439***	-0.578***	-0.556***	-0.601***
**PET**				-0.322***	-0.674***	-0.662***	-0.731***
**PDQ**					0.343***	0.176*	0.345***
**MAP**						0.982***	0.971***
**PWQ**							0.954***

Significant levels: *, $p < 0.05$; **, $p < 0.01$; ***, $p < 0.001$.

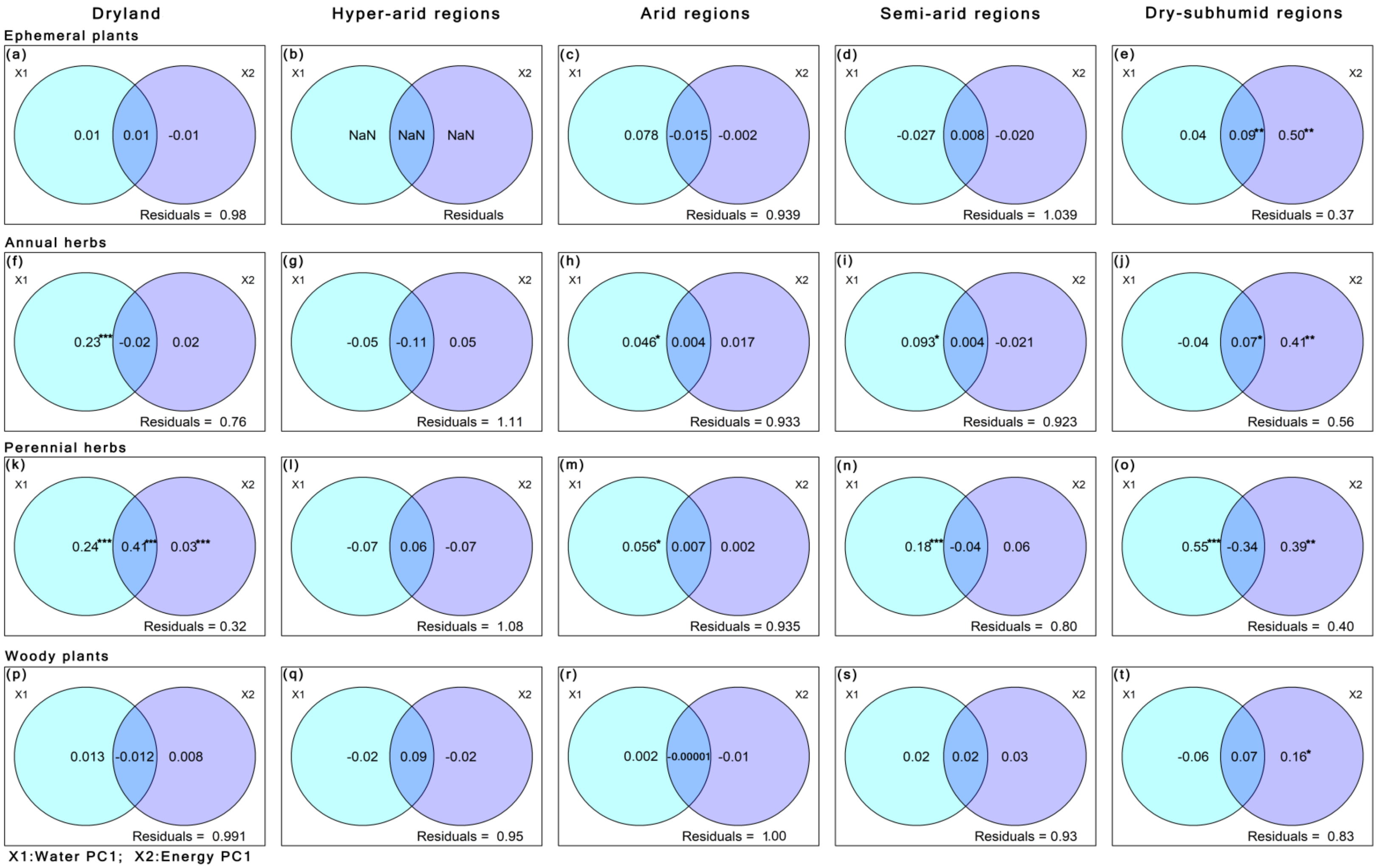

Figure S1. The variation of water and energy effects on plant diversity for life-forms by variation partitioning. The left value is the explained variability by the independent components of water; the middle value represents variability explained by the co-varying component of water and energy, the right value is the explained variability by the independent components of environmental. Residuals are representing unexplained variability. All of the variation values are adjusted by coefficient of determination. In Figure S1 (b): NaN represents the only three sites for ephemeral plants in hyper-arid regions, so it is not operable by variation partitioning.
